# CO_2_‐to‐CO Electrochemical Conversion With an Fe(I) Porphyrin Complex in Water

**DOI:** 10.1002/anie.7585384

**Published:** 2026-08-05

**Authors:** Andrew Howe, Aude Salamé, Ulysse Garnier, Marc Robert, Elodie Anxolabéhère‐Mallart, Mun Hon Cheah

**Affiliations:** ^1^ Molecular Biomimetics Department of Chemistry‐Ångström Uppsala University Uppsala Sweden; ^2^ Leiden Institute of Chemistry Leiden University Leiden, RA the Netherlands; ^3^ Institut Parisien de Chimie Moléculaire (IPCM) CNRS Sorbonne Université Paris France; ^4^ Institut Universitaire de France (IUF) Paris France

**Keywords:** CO_2_ reduction, iron porphyrin, mechanism, molecular catalysis, spectroelectrochemistry

## Abstract

Iron porphyrin complexes constitute a well‐established and versatile class of molecular electrocatalysts for the reduction of CO_2_ to CO. In both organic and aqueous media, the reaction mechanism is typically proposed to involve the interaction of CO_2_ with a formally defined [(porphyrin)Fe^0^] intermediate. In this work, we performed a mechanistic investigation of CO_2_ reduction using the water‐soluble complex [(*p*TMA)Fe^III^Cl]Cl_4_ under aqueous conditions. In situ scanning spectroelectrochemistry was employed, enabling the synchronized acquisition of UV–vis or IR spectra during cyclic voltammetry experiments. Our results provide strong evidence for CO_2_ binding to the electrogenerated [(*p*TMA)Fe^I^]^3+^ species, followed by reductive C─O bond cleavage to yield a stable [(*p*TMA)(Cl)Fe^II^‐CO]^3^^+^ complex. This process corresponds to an overall two‐electron reduction per iron center. This mechanism, which has not been previously considered for molecular iron porphyrins in CO_2_ reduction, is proposed to be facilitated by the charged porphyrin periphery and the hydrogen‐bonding network of the aqueous medium. These features may open new avenues toward achieving CO_2_ reduction at lower overpotentials in water.

## Graphical abstract

Using in situ UV–vis/IR spectroelectrochemistry, we track CO_2_ reduction by a water‐soluble iron porphyrin. Rather than the usual [Fe^0^] intermediate in organic solvents, CO_2_ binds the electrogenerated [Fe^I^] species and undergoes reductive C─O cleavage to stable [Fe^II^‐CO]. This previously unexplored reaction pathway is facilitated by an aqueous H‐bonding network and charged periphery groups of iron porphyrins.

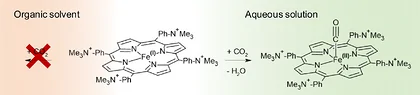

## 1 Introduction

Iron porphyrin complexes are a well‐known class of molecular catalysts for performing CO_2_ reduction to CO with high selectivity in organic solvents such as DMF [1, 2, 3, 4, 5, 6]. The CO_2_ reduction mechanism of these complexes is primarily based on experimental mechanistic studies conducted in organic solvents and supplemented by computational investigations and complementary spectroscopic insights [7, 8]. CO_2_ binds to the iron porphyrin at the formal [(porphyrin)Fe^0^] oxidation level, resulting in a [(porphyrin)Fe^II^‐CO_2_] species. Subsequent protonation steps and dehydration result in a [(porphyrin)Fe^II^‐CO] species, which is further reduced by one electron before decarbonylation to close the catalytic cycle [2]. The exact sequencing of electron and proton transfer after CO_2_ binding depends on the substituent of the phenyl ligand. For example, in the case of FeTPP, protonation of the CO_2_‐bound adduct is concerted with C─O bond cleavage, generating an Fe^II^‐CO species that is further reduced to Fe^I^‐CO and decoordinates CO. In contrast, with the porphyrin substituted with an OH group in all *ortho*, *ortho’* positions of the phenyl rings, protonation of the Fe‐CO_2_ adducts (forming a stabilized Fe‐COOH species) precedes electron transfer, which is necessary to cleave the C─O bond through a concerted process [9].

This mechanistic scheme, derived from studies in organic solvents, is often used to rationalise CO_2_ reduction in aqueous conditions by water‐soluble or heterogenised iron porphyrins. However, direct translation of mechanistic insights derived from organic solvents to aqueous systems for CO_2_ reduction is complicated by differences in solvent, counter ions, proton donors, H‐bond partners and even speciation of dissolved CO_2_ due to hydration and acid‐base equilibria [10]. To date, there are limited experimental mechanistic studies of CO_2_ reduction in aqueous conditions [11, 12], and the interpretation of these studies is usually based on the mechanistic framework established in organic solvents above.

In this study, we conducted a mechanistic study of CO_2_ reduction by the well‐known water‐soluble meso‐tetra(4‐N,N, N‐trimethylanilinium) iron (III) porphyrin chloride, which is denoted as [(*p*TMA)Fe^III^Cl]^4+^ (Scheme 1). [(*p*TMA)Fe^III^Cl]^4+^ was reported to undergo three successive one‐electron reductions, observed as three well‐separated reversible redox waves in cyclic voltammetry in DMF solvent [3]. Reduction of CO_2_ is reported to proceed at the formal [(*p*TMA)Fe^0^]^2+^ oxidation state [3], and we have recently added new mechanistic insights by complementary spectroelectrochemical studies [13]. In aqueous solution, reduction of CO_2_ was reported to occur at the formal [(*p*TMA)Fe^0^]^2+^ state, being in competition with the proton reduction reaction [12]. In addition to electrochemical reduction of CO_2_, [(*p*TMA)Fe^III^Cl]^4+^ has also been shown to perform photochemical CO_2_ reduction in both organic solvents and aqueous conditions [14, 15, 16, 17]. Using the scanning spectroelectrochemistry (SEC) method, UV–vis and IR spectra were collected during cyclic voltammetry experiments, evidencing CO_2_ binding and reduction at the formal [(*p*TMA)Fe^I^]^3+^ oxidation state under aqueous conditions. CO_2_ coordination to the Fe(I) electrogenerated species is followed by the reductive cleavage of one C─O bond leading to a stable Fe^II^‐CO species, in an overall 2‐electron per Fe center reduction wave.

**SCHEME 1:**
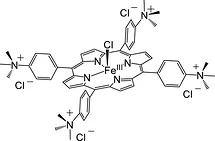
Structure of [(*p*TMA)Fe^III^Cl]^4+^.

## 2 Results and Discussion

### 2.1 Reduction of [(*p*TMA)Fe^III^Cl]^4+^ Under Ar Atmosphere

First, we clarified the speciation of [(*p*TMA)Fe^III^Cl]^4+^ in aqueous medium at open circuit potential (OCP), as this has been overlooked in previous mechanistic studies [7, 11, 12]. It has been reported that the axial chloride ligand in [(*p*TMA)Fe^III^Cl]^4+^ is displaced in aqueous buffer or electrolytes, resulting in the formation of a *μ*‐oxo dimer, (*μ*‐O[(*p*TMA)Fe^III^]_2_)^8+^ [18]. Comparison of UV–vis spectrum of [(*p*TMA)Fe^III^Cl]^4+^ in this study to that reported in DMF [13] showed clear differences in the position of the Soret and Q bands (Figure 1). The observed spectrum in aqueous medium in this study is consistent with the reported spectrum of the *μ*‐oxo dimer, (*μ*‐O[(*p*TMA)Fe^III^]_2_)^8+^, indicating that this is the species present at OCP.

**FIGURE 1:**
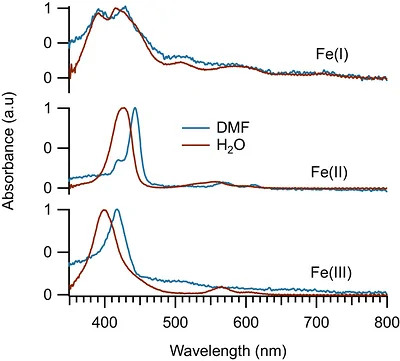
Comparison of UV–vis spectra of [(*p*TMA)Fe] species recorded in DMF (cyan) and aqueous conditions, 0.2 M phosphate (pH = 7), 0.2 M KCl (brown). The spectra recorded in DMF were published in a previous work [13]. The spectra recorded in aqueous conditions are reproduced from Figure 2.

We investigated the reduction of [(*p*TMA)Fe^III^Cl]^4+^ dissolved in aqueous medium (0.2 M phosphate buffer, pH = 7.0, 0.2 M KCl), forming (*μ*‐O[(*p*TMA)Fe^III^]_2_)^8+^, under Ar atmosphere up to the second reduction wave corresponding to the generation of a Fe(I) species (Figure 2A). The reduction of (*μ*‐O[(*p*TMA)Fe^III^]_2_)^8+^ to [(*p*TMA)Fe^II^]^4+^ appeared to be a kinetically slow process, as there was no well‐defined reduction peak in the cyclic voltammogram (Figure 2A). However, this reduction process could be conveniently monitored by changes in the UV–vis spectra as a function of applied potential, revealing a slow conversion rate which is consistent with the observed current response (Figure 2A,B). These slow reduction kinetics were visualised by plotting the derivative of the normalised (*μ*‐O[(*p*TMA)Fe^III^]_2_)^8+^ population as a function of the applied potential (Figure 2A), where the reduction of this Fe^III^ species appeared as a broad feature. Upon reduction of (*μ*‐O[(*p*TMA)Fe^III^]_2_)^8+^, we hypothesize that [(*p*TMA)Fe^II^]^4+^ exists in monomeric form based on precedence in the literature for related water‐soluble iron porphyrin complexes [19, 20, 21]. The presence of (*μ*‐O[(*p*TMA)Fe^III^]_2_)^8+^ as a *μ*‐oxo dimer, while [(*p*TMA)Fe^II^]^4+^ remains monomeric, may rationalise the observation that reduction of (*μ*‐O[(*p*TMA)Fe^III^]_2_)^8+^ in aqueous conditions appeared to be kinetically slow compared to reduction of [(*p*TMA)Fe^II^]^4+^ to [(*p*TMA)Fe^I^]^3+^ and oxidation of [(*p*TMA)Fe^II^]^4+^. A second reversible redox process was observed at −0.92 V versus Ag/AgCl, corresponding to the reduction of [(*p*TMA)Fe^II^]^4+^ to [(*p*TMA)Fe^I^]^3+^. The UV–vis spectrum of [(*p*TMA)Fe^I^]^3+^ was similar to that reported in DMF solvent [13].

**FIGURE 2:**
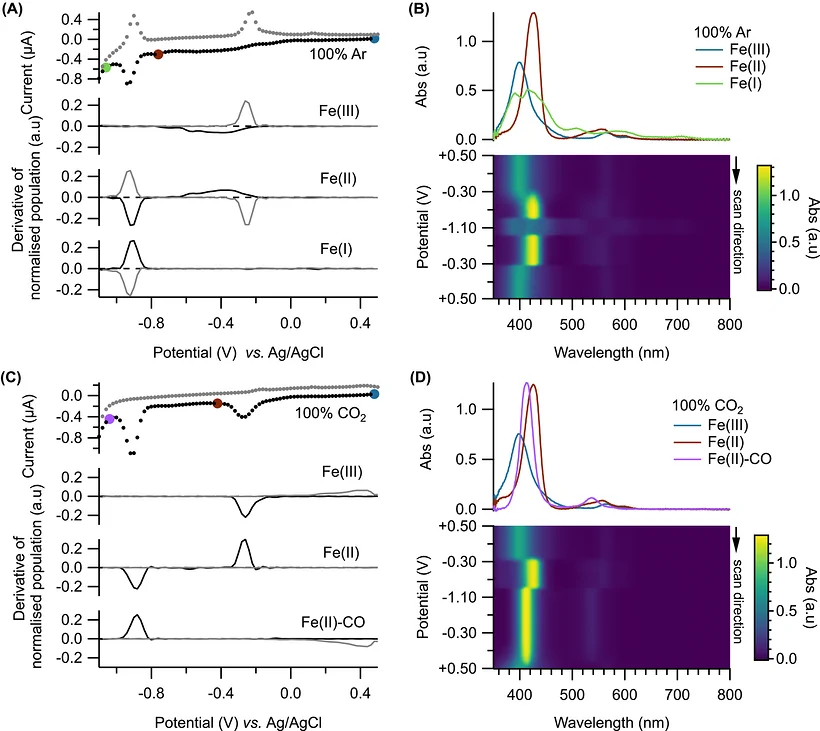
UV–vis SEC data of 2 mM solution of [(*p*TMA)Fe^III^Cl]Cl_4_ dissolved in 0.2 M potassium phosphate buffer + 0.2 M KCl; see text for speciation of [(*p*TMA)Fe^III^Cl]Cl_4_. The potential step was 20 mV and the average scan rate 1 mV/s. Panel (A) and (B): 3 bar 100% Ar, pH = 7; panel (C) and (D): 3 bar 100% CO_2_, pH ≈ 6. Top panel of (A) and (C): Current‐voltage response during UV–vis SEC, cathodic scan direction (black) and anodic scan direction (gray). Bottom panel of (A) and (C): Derivative of normalised population of [(*p*TMA)Fe] species, from deconvolution of the UV–vis spectra. Top panel of (B) and (D): Representative spectra recorded during UV–vis SEC where the applied potential was color coded based on the matching color of the large circular symbol in the corresponding panel (A) and (C). Bottom panels of (B) and (D) are 2D‐image plots of UV–vis spectra as a function of the applied potential.

On the reverse anodic scan, a well‐defined reoxidation peak at −0.25 V versus Ag/AgCl was obtained, corresponding to the oxidation of [(*p*TMA)Fe^II^]^4+^ to (*μ* ‐O[(*p*TMA)Fe^III^]_2_)^8+^. We observed full recovery of the starting material of (*μ*‐O[(*p*TMA)Fe^III^]_2_)^8+^, based on the observed UV–vis spectra at the end of the SEC scan (Figure 2B, 2D‐image plot at bottom). This is consistent with the well‐known reversible first and second reduction of [(*p*TMA)Fe^III^Cl]^4+^ in the literature [13]. Based on these observations, we conclude that the conversion between (*μ*‐O[(*p*TMA)Fe^III^]_2_)^8+^ and [(*p*TMA)Fe^II^]^4+^ is a multi‐step process, that is, kinetically slow but chemically reversible.

### 2.2 Reduction of [(*p*TMA)Fe^III^Cl]^4+^ Under CO_2_ Atmosphere

When we examined the reduction of (*μ*‐O[(*p*TMA)Fe^III^]_2_)^8+^ under 3 bar of CO_2_ atmosphere in potassium phosphate buffer (final measured pH ≈ 6) (Figure 2C,D), we observed very different current and spectral response as compared to Ar (Figure 2A,B). Such observation is significant, as [(*p*TMA)Fe^II^]^4+^ and [(*p*TMA)Fe^I^]^3+^ were reported not to react with CO_2_ in organic solvents such as DMF, and postulated CO_2_ reduction mechanisms in water as solvent are based on those derived in organic solvents [7, 11, 12, 13].

Under a CO_2_ atmosphere, we observed a well‐defined reduction wave at −0.26 V versus Ag/AgCl, corresponding to the conversion of (*μ*‐O[(*p*TMA)Fe^III^]_2_)^8+^ to [(*p*TMA)Fe^II^]^4+^ based on changes in the UV–vis spectra. The UV–vis spectrum of the species at OCP under Ar and CO_2_ appeared identical. Similarly, the UV–vis spectrum of [(*p*TMA)Fe^II^]^4+^ observed under CO_2_ was very similar to that observed under Ar (Figure 2B,D), suggesting little or no reaction between [(*p*TMA)Fe^II^]^4+^ and CO_2_. When the SEC scan was reversed before the second reduction wave, (*μ*‐O[(*p*TMA)Fe^III^]_2_)^8+^ was quantitatively recovered from [(*p*TMA)Fe^II^]^4+^ (Figure S1). We postulate that the observed differences in voltammograms between Ar and CO_2_ atmospheres are likely due to differences in the pKa of available proton donors (H_2_PO_4_^−^, pKa = 7.2 under Ar vs. H_2_CO_3_, pKa = 3.5 under CO_2_), as reduction of (*μ*‐O[(*p*TMA)Fe^III^]_2_)^8+^ requires two protonation steps to break a *μ*‐O bridge.

A second reduction wave appeared at −0.90 V versus Ag/AgCl, with peak current intensity ca. 1.9 times that of the first reduction peak at −0.26 V versus Ag/AgCl. The integrated peak current for the second reduction wave under CO_2_ was indeed almost two times larger than that observed under an Ar atmosphere (Figure S2). This indicates a two‐electron irreversible reduction process, as evidenced by the lack of a reoxidation peak upon anodic scan. Most interestingly, the UV–vis spectrum of the product of this two‐electron reduction of [(pTMA)Fe^II^]^4+^ was almost identical to that of [(*p*TMA)(Cl)Fe^II^‐CO]^3+^ (see IR‐SEC below regarding Cl^−^ coordination). The assignment of this spectrum to [(*p*TMA)(Cl)Fe^II^‐CO]^3+^ was verified by comparison to a SEC scan conducted under 50% CO and 50% CO_2_ atmosphere, as it is well documented that [(*p*TMA)Fe^II^]^4+^ reacts with CO to form [(*p*TMA)(Cl)Fe^II^‐CO]^3+^ [13]. After the first reduction wave (−0.24 V vs. Ag/AgCl) in the presence of CO, we did not obtain the spectrum of [(*p*TMA)Fe^II^]^4+^ (Figure 3, blue trace), instead, a new spectrum that can be assigned as [(*p*TMA)(Cl)Fe^II^‐CO]^3+^ was formed and almost identical to that observed when [(*p*TMA)Fe^III^Cl]^4+^ was reduced to the Fe^I^ oxidation state under 100% CO_2_ (Figure 3, red trace).

**FIGURE 3:**
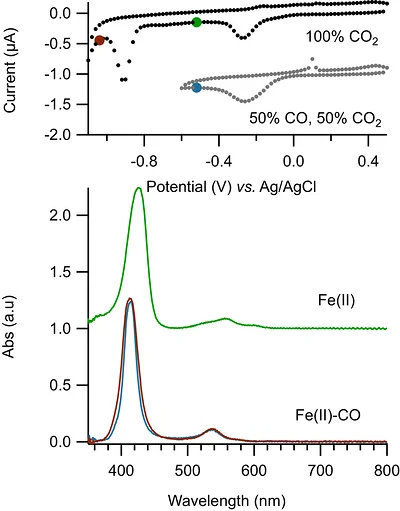
UV–vis SEC data of 2 mM solution of [(*p*TMA)Fe^III^Cl]Cl_4_ dissolved in 0.2 M potassium phosphate buffer + 0.2 M KCl. The potential step was 20 mV and the average scan rate 1 mV/s. Top panel: Current‐voltage response recorded during UV–vis SEC, 3 bar 100% CO_2_ (black) and 3 bar 50% CO_2_ and 50% CO (gray). Bottom panel: UV–vis spectra recorded during UV–vis SEC, color coded to match the filled circle in the top panel, offset for clarity.

The formation of [(*p*TMA)(Cl)Fe^II^‐CO]^3+^ under a CO_2_ atmosphere was further verified by IR‐SEC experiments (0.2 M phosphate buffer pH ≈ 6, no exogenous KCl) (Figure 4). Upon reduction at the second reduction wave (−0.90 V vs. Ag/AgCl), a vibrational band with an apparent peak at 1972 cm^−1^ was formed, indicative of a metal carbonyl species, tentatively assigned as [(*p*TMA)Fe^II^‐CO]^4+^. On closer inspection, this ν(CO) band is asymmetric and could be deconvoluted into two bands centred at 1973 and 1962 cm^−1^, respectively. Analogous experiments conducted in the presence of 200 mM KCl only resulted in a single band centred at 1962 cm^−1^ (Figure S3). Based on these observations, we can rationalise the assignment of the 1962 cm^−1^ band to [(*p*TMA)(Cl)Fe^II^‐CO]^3+^ with Cl^−^ bound trans to the CO ligand, while the 1973 cm^−1^ band is assigned to [(*p*TMA)Fe^II^‐CO]^4+^ without bound Cl^−^. In experiments without exogenous KCl, there was a mixture of the two species since there are 5 equivalents of Cl^−^ present in the parent complex. We previously reported the same band at 1962 cm^−1^, assigned as [(*p*TMA)Fe^II^‐CO]^4+^ in experiments conducted in DMF without exogenous chloride [13]. This assignment should be revised as [(*p*TMA)(Cl)Fe^II^‐CO]^3+^ in light of the present observations.

**FIGURE 4:**
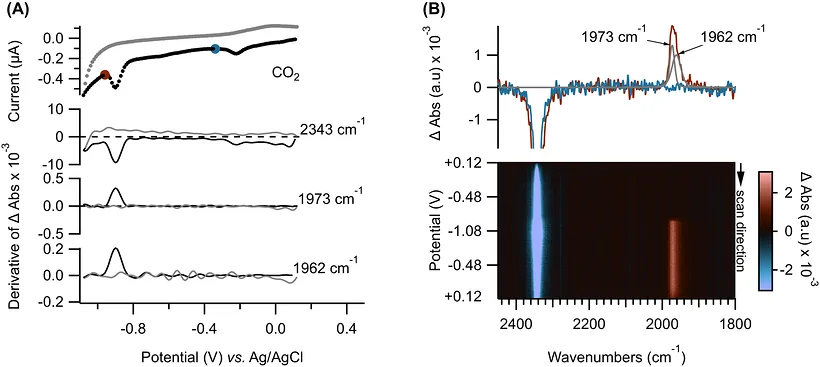
IR SEC data of 2 mM of [(*p*TMA)Fe^III^Cl]Cl_4_ in 0.2 M potassium phosphate buffer under 3 bar 100% CO_2_ (pH ≈ 6). The potential step was 20 mV and the average scan rate 1 mV/s. Top panel of (A) Current‐voltage response during IR SEC, cathodic scan direction (black) and anodic scan direction (gray). Bottom panel of (A) Derivative of peak intensities of ν(CO) bands, from deconvolution of IR spectra. Top panel of (B) Representative spectra recorded during IR SEC where the applied potential is color coded based on the matching color of the large circular symbol in the corresponding panel (A). Bottom panels of (B) 2D‐image plot of IR spectra as a function of the applied potential.

At the same time, we observed a corresponding depletion of peak intensity at 2343 cm^−1^ that can be assigned to asymmetric ν(CO) of dissolved CO_2_. Taken together, these observations strongly suggest CO_2_ binding to [(*p*TMA)Fe^I^]^3+^ and subsequent reduction to CO to produce [(*p*TMA)Fe^II^‐CO]^4+^.

### 2.3 HCO_3_^−^ as a Substrate for Reduction to CO?

The hydration of CO_2_ and water produces H_2_CO_3_ and then HCO_3_^−^ in acid‐base equilibrium reactions, raising the possibility that HCO_3_^−^ may be a substrate for CO production upon binding to the reduced porphyrin. Based on DFT calculations of a simple model iron porphyrin, Khakpour and coworkers proposed that reduction of HCO_3_^−^ and H_2_CO_3_ is kinetically and thermodynamically favorable, while reduction of CO_2_ is kinetically hindered at the formal [(porphyrin)Fe^I^] oxidation state [22].

Here we examined this hypothesis by comparing the reduction of (*μ*‐O[(*p*TMA)Fe^III^]_2_)^8+^ in bicarbonate buffer under Ar and CO_2_ atmospheres (Figure 5). Under an Ar atmosphere, in the presence of 0.2 M bicarbonate buffer adjusted to pH 7, the CV of (*μ*‐O[(*p*TMA)Fe^III^]_2_)^8+^ appeared different from that recorded in phosphate buffer at pH 7. A redox wave, assigned to reduction of (*μ*‐O[(*p*TMA)Fe^III^]_2_)^8+^ to [(*p*TMA)Fe^II^]^4+^ based on observed UV–vis spectra, was seen at −0.72 V versus Ag/AgCl. A plausible rationalization of this shift in reduction potential of (*μ*‐O[(*p*TMA)Fe^III^]_2_)^8+^ may be related to the pK_a_ and concentration of the proton donor present in solution, as reduction of (*μ*‐O[(*p*TMA)Fe^III^]_2_)^8+^ to [(*p*TMA)Fe^II^]^4+^ involves double protonation of the *μ*‐O bridge.

**FIGURE 5:**
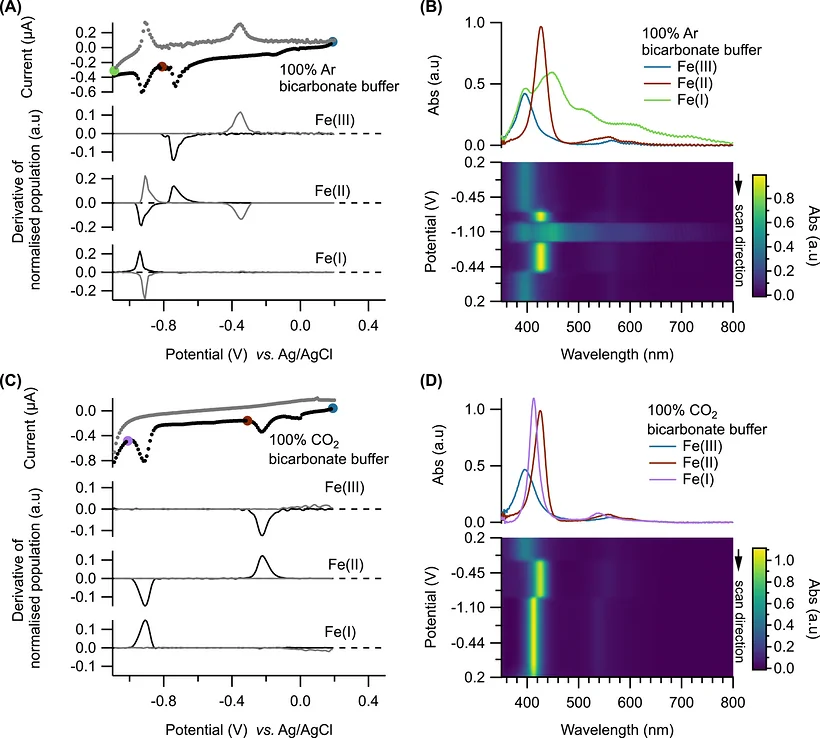
UV–vis SEC data of 2 mM solution of [(*p*TMA)Fe^III^Cl]^4+^ in 0.2 M sodium bicarbonate buffer + 0.2 M KCl. The potential step was 20 mV and the average scan rate 1 mV/s. Panel (A) and (B): 3 bar 100% Ar, pH = 7; panel (C) and (D): 3 bar 100% CO_2_, pH ≈ 6. Top panel of (A) and (C): current‐voltage response during UV–vis SEC, cathodic scan direction (black) and anodic scan direction (gray). Bottom panel of (A) and (C) Derivative of normalised population of [(*p*TMA)Fe] species, from deconvolution of UV–vis spectra. Top panel of (B) and (D) Spectra recorded during UV–vis SEC where the applied potential is color coded based on the matching color of the large circular symbol in the corresponding panel (A) and (C). Bottom panels of (B) and (D) are 2D‐image plots of UV–vis spectra as a function of applied potential.

This wave was closely followed by a reversible redox wave at −0.91 V versus Ag/AgCl, corresponding to the [(*p*TMA)Fe^II^]^4+^/[(*p*TMA)Fe^I^]^3+^ redox couple. The current response of this redox couple appeared very sharp, suggesting some deposition of an insoluble [(*p*TMA)Fe^I^]^3+^ species. This is consistent with the relatively sharp feature observed in the corresponding spectra‐potential response. Moreover, the overall background of the UV–vis spectrum of [(*p*TMA)Fe^I^]^3+^ appeared elevated, which is consistent with formation of an insoluble material on the electrode surface, lowering its reflectivity and thereby causing an apparent rise in baseline absorption. On the anodic scan, there was an oxidation wave at −0.35 V versus Ag/AgCl, assigned to oxidation of [(*p*TMA)Fe^II^]^4+^ to (*μ*‐O[(*p*TMA)Fe^III^]_2_)^8+^ based on the observed UV–vis spectra.

While the reduction potential and kinetics of (*μ*‐O[(*p*TMA)Fe^III^]_2_)^8+^ in bicarbonate buffer were different as compared to phosphate buffer, the potential of the [(*p*TMA)Fe^II^]^4+^/[(*p*TMA)Fe^I^]^3+^ redox couple appeared unchanged (−0.90 V in phosphate buffer vs. −0.91 V in bicarbonate buffer), suggesting similar speciation of [(*p*TMA)Fe^II^] in both buffer conditions. More importantly, there was no evidence of reaction between [(*p*TMA)Fe^I^]^3+^ and bicarbonate under Ar atmosphere to form [(*p*TMA)(Cl)Fe^II^‐CO]^3+^, therefore ruling out HCO_3_^−^ as substrate for reduction to CO (Figure 5A,B).

Under a CO_2_ atmosphere in bicarbonate buffer, the current and spectral response was similar to that observed in phosphate buffer. A reduction peak at −0.22 V versus Ag/AgCl was obtained, corresponding to the conversion of (*μ*‐O[(*p*TMA)Fe^III^]_2_)^8+^ to [(*p*TMA)Fe^II^]^4+^, followed by a second reduction at −0.92 V versus Ag/AgCl, with approximately twice the peak current intensity when compared to the first reduction peak. The spectra recorded after the second reduction wave can be assigned to [(*p*TMA)(Cl)Fe^II^‐CO]^3+^, indicating CO_2_ binding to [(*p*TMA)Fe^I^]^3+^ and subsequent reduction to form [(*p*TMA)(Cl)Fe^II^‐CO]^3+^.

## 3 Discussion

In this study, we identified the reaction between [(*p*TMA)Fe^I^]^3+^ and CO_2_ to form a [(*p*TMA)Fe^II^‐CO]^4+^ species in aqueous buffer electrolyte. Based on the UV–vis and IR SEC data presented above, we propose the following reaction sequence at the second reduction wave:

$${\left[\left(p\mathrm{TMA}\right)Fe^{\mathrm{II}}\right]}^{4+}+e^{-}\;\to \;{\left[\left(p\mathrm{TMA}\right)Fe^{I}\right]}^{3+}$$

$${\left[\left(p\mathrm{TMA}\right)Fe^{I}\right]}^{3+}+\;CO_{2}\;\to \;{\left[\left(p\mathrm{TMA}\right)Fe^{\mathrm{III}}-CO_{2}\right]}^{3+}$$

$${\left[\left(p\mathrm{TMA}\right)Fe^{\mathrm{III}}-CO_{2}\right]}^{3+}+H^{+}\;\to \;{\left[\left(p\mathrm{TMA}\right)Fe^{\mathrm{III}}-\mathrm{COOH}\right]}^{4+}$$

$${\left[\left(p\mathrm{TMA}\right)Fe^{\mathrm{III}}-\mathrm{COOH}\right]}^{4+}+e^{-}\;\to \;{\left[\left(p\mathrm{TMA}\right)Fe^{\mathrm{II}}-\mathrm{COOH}\right]}^{3+}$$

$${\left[\left(p\mathrm{TMA}\right)Fe^{\mathrm{II}}-\mathrm{COOH}\right]}^{3+}+H^{+}\;\to \;{\left[\left(p\mathrm{TMA}\right)Fe^{\mathrm{II}}-\mathrm{CO}\right]}^{4+}+H_{2}O$$

This ECCEC sequence of reactions includes the reduction of [(*p*TMA)Fe^II^]^4+^ to [(*p*TMA)Fe^I^]^3+^, oxidative addition of CO_2_ to [(*p*TMA)Fe^I^]^3+^, subsequent protonation, reduction, and dehydration to form [(*p*TMA)Fe^II^‐CO]^4+^, resulting in an overall two‐electron reduction process. We note that the precise sequencing of protonation and reduction following oxidative addition of CO_2_ to [(*p*TMA)Fe^I^]^3+^ cannot be resolved by the current data and is rather postulated based on simple chemical reasoning, as protonation of [(*p*TMA)Fe^II^‐COOH]^3+^ is likely more favourable compared to protonation of [(*p*TMA)Fe^III^‐COOH]^4+^. Notably, concerted proton‐coupled electron transfer to [(*p*TMA)Fe^III^‐COOH]^4+^ may occur. In addition, Cl^−^ ligation of [(*p*TMA)Fe^II^‐CO]^4+^ to form the end product [(*p*TMA)(Cl)Fe^II^‐CO]^3+^ is not included in this proposed sequence.

The reduction of [(*p*TMA)(Cl)Fe^II^‐CO]^3+^ is expected to be approximately 200 mV more cathodic than [(*p*TMA)Fe^II^]^4+^, based on corresponding observation in DMF solvent [13]. As such, we did not observe catalytic turnover of CO_2_ within the potential window of the experiments in this study. To confirm this absence of catalysis, we performed a bulk electrolysis experiment with 2 mM of [(*p*TMA)Fe^III^Cl]^4+^ in 0.2 M phosphate buffer (pH = 7) with 0.2 M KCl under 1 bar of CO_2_. After 3 h and the transfer of slightly more than 3 electrons per Fe porphyrin molecule, no CO nor H_2_ could be detected by GC (Figure S4), confirming that the CO remains bound to the Fe porphyrin.

The reaction between [(*p*TMA)Fe^I^]^3+^ and CO_2_ in the presence of aqueous buffer electrolyte is in stark contrast to a previous report in DMF solvent, where [(*p*TMA)Fe^I^]^3+^ does not react with CO_2_. A possible rationalization of this reactivity could be the presence of a strong σ‐donating axial ligand coordinating to the Fe center of [(*p*TMA)Fe^I^]^3+^, increasing the electron density of the Fe center to facilitate CO_2_ coordination. This hypothesis can be examined by comparison of the UV–vis spectra collected in aqueous and DMF conditions, where it can be seen that the Soret and Q bands are highly sensitive to the identity of axial ligands (Figure 1). As noted earlier, the speciation of [(*p*TMA)Fe^III^Cl]^4+^ dissolved in DMF and aqueous medium is different due to dimer formation of (*μ*‐O[(*p*TMA)Fe^III^]_2_)^8+^. The position of the Soret and Q bands of [(*p*TMA)Fe^II^] in aqueous conditions is blue‐shifted compared to that recorded in DMF solvent. This observation is consistent with literature reports where axial ligands of iron porphyrins in formal Fe^II^ oxidation state are highly dependent on the solvation environment and presence of competing axial ligands [23, 24, 25, 26]. Most importantly and in contrast, the positions of the Soret and Q bands of [(*p*TMA)Fe^I^]^3+^ in both aqueous and DMF solvents are very similar. This suggests that the axial ligands of [(*p*TMA)Fe^I^]^3+^ are indeed identical, or more likely, that there are no axial ligands present for [(*p*TMA)Fe^I^]^3+^ [23, 26, 27]. Therefore, although axial ligation may be beneficial, its influence on the reactivity of [(*p*TMA)Fe^I^]^3+^ towards CO_2_ is insufficient to account for the experimental observations.

It has been proposed in previous studies that the reduction of [(*p*TMA)Fe^II^]^4+^ under a CO_2_ atmosphere in aqueous media can be accompanied by protonation of the porphyrin N atom of *p*TMA [7, 11]. The proton source for this reaction was proposed to be carbonic acid, H_2_CO_3_, obtained from the hydration of dissolved CO_2_. Based on DFT calculations, Zhang and coworkers proposed that protonation of the porphyrin N atom provides a lower‐energy pathway towards a formal Fe^0^ species for CO_2_ binding and subsequent reduction [7]. Similarly, Li and coworkers reported protonation of the porphyrin N atom during reduction of [(*p*TMA)Fe^II^]^4+^ based on IR‐SEC experiments [11]. While both studies postulated CO_2_ binding to the formal [(*p*TMA)Fe^0^]^2+^ species, it is conceivable that a protonated N species of [(*p*TMA)Fe^I^]^3+^ may facilitate CO_2_ binding. However, as noted in the DFT studies above [7], CO_2_ binding to an N‐protonated [(*p*TMA)Fe^0^] ^2+^ species is endergonic by 13.5 kcal/mol, while CO_2_ binding to the non‐protonated species is slightly exergonic by 1.8 kcal/mol. This can be rationalised by the increased delocalisation of electron density to the protonated porphyrin ring, thereby lowering the nucleophilicity of the Fe center towards the empty LUMO of CO_2_. This concept of increasing electron delocalization to the protonated porphyrin ring stands in line with experimental and computational studies of Römelt and coworkers [23], where reduction of the closely related iron(II) tetraphenylporphyrin, [(TPP)Fe^II^], was proposed to be predominantly ligand‐centered to yield [(TPP^•^)Fe^II^]^1−^ and [(TPP^••^)Fe^II^]^2−^. Upon CO_2_ binding to [(TPP^••^)Fe^II^], it was postulated that the electron density on the porphyrin ring would shift toward the activated CO_2_ [8]. For the [(*p*TMA)Fe^I^]^3+^ species, we may expect a similar phenomenon to prevail, namely, protonation of the porphyrin N atom with delocalized electron density towards the porphyrin ring, thus decreasing nucleophilicity of the Fe center towards CO_2_. As such, we do not expect that the protonation of the porphyrin N atom of [(*p*TMA)Fe^I^] would enhance CO_2_ binding. In this connection, we note that contrary to the report of Li and coworkers [11], we did not observe the formation of a band at 2538 cm^−1^, which was assigned by these authors to an N─H^+^ vibration to support protonation of the porphyrin N atom (Figure S5).

Based on the preceding discussions, it appears that the reaction between CO_2_ and the Fe^I^ center may alternatively originate from solvation effects, possibly enhanced with second coordination sphere interactions. We propose that CO_2_ binding to [(*p*TMA)Fe^I^]^3+^ is due to stabilisation of [(*p*TMA)Fe^III^‐CO_2_]^3+^ species by an extensive hydrogen bonding network provided by the aqueous buffer environment, via solvent, buffer, or combination of the two, possibly combined with additional solvation structuration provided by the four trimethylammonium groups. It is also possible that this adduct species may be additionally stabilised by axial ligation to the formal Fe^III^ center once CO_2_ is coordinated, either as a concerted step with CO_2_ binding or as a subsequent reaction after CO_2_ binding (Scheme 2), even if this stabilisation is not able alone to trigger the reactivity. We conducted analogous experiments with the negatively charged metalloporphyrin [(TPPS)Fe^III^Cl] (TPPS = 5,10,15,20‐Tetrakis(4‐sulfonatophenyl)porphyrin) and observed similar reactivity at the Fe^I^ center (Figures S6 and S7). On the other hand, experiments with the neutral porphyrin [(TPP)Fe^III^Cl] in DMF with 200 mM H_2_O do not show any reaction between CO_2_ and Fe^I^ centers (Figure S8). All these observations stand in line with our hypothesis that a specific strong hydrogen bonding network is required for enabling reaction between Fe^I^ species and CO_2_. Further spectroscopic studies may shed more light on the exact role of this strong hydrogen bonding network, which is likely facilitated by the presence of charged periphery groups. A related observation was reported by Amanullah et al., where an iron porphyrinoid, with a pendant amine moiety that provides a second coordination sphere H‐bonding, is proposed to reduce CO_2_ from the Fe(I) oxidation state to formate in organic solvent [28].

**SCHEME 2:**
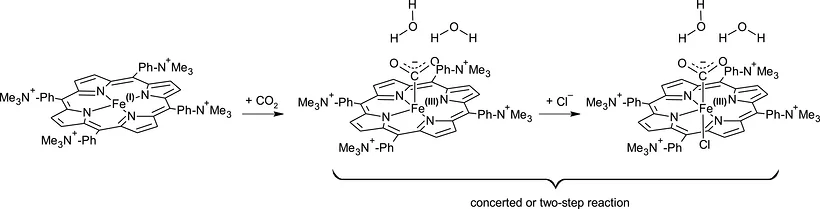
Proposed CO_2_ binding to [(*p*TMA)Fe^I^]^3+^ in aqueous conditions. Binding sequence can either occur as a concerted or a two‐step reaction. The identity of the axial ligand Cl^−^ is postulated based on the formation of [(*p*TMA)(Cl)Fe^II^‐CO]^3+^ as a stable reaction product. Note that the molecules providing hydrogen bonding and the associated network geometry to [(*p*TMA)Fe^III^‐CO_2_]^3+^ are unknown. It is depicted here with two water molecules as a generic representation of an extensive H‐bonding network.

## 4 Conclusion

Upon the combination of UV–vis and IR SEC experiments, CO_2_ binding to [(*p*TMA)Fe^I^]^3+^ and subsequent reduction to form [(*p*TMA)(Cl)Fe^II^‐CO]^3+^ in aqueous experimental conditions has been identified. This pathway is viable for metalloporphyrins in aqueous solvent that provides an extensive H‐bonding network to stabilize the CO2‐bound intermediate and may be further helped with charged periphery groups on the ligand framework. This study thus revealed a previously unexplored pathway for CO_2_ reduction operating at the formal Fe^I^ oxidation state, yielding [(*p*TMA)(Cl)Fe^II^‐CO]^3+^. We did not observe catalytic turnover of CO_2_ into CO by [(*p*TMA)Fe] at applied potential corresponding to formation of [(*p*TMA)Fe^I^]^3+^, due to an inhibited CO dissociative pathway to close the catalytic cycle. While applying a more cathodic reduction potential will result in reduction of [(*p*TMA)(Cl)Fe^II^‐CO]^3+^ and close the catalytic cycle, this will also result in further reduction to the Fe^0^ oxidation level. As an alternative future strategy, it may be possible for photolysis, under appropriate conditions, of [(*p*TMA)(Cl)Fe^II^‐CO]^3+^ to decarbonylate CO [29] and generate [(*p*TMA)Fe^II^]^4+^ to close the catalytic cycle. Such a strategy would result in lower overpotential for CO_2_ reduction to CO and bypass competing H^+^ reduction pathways, thus opening new opportunities for using molecular iron porphyrin complexes for CO_2_ reduction in aqueous systems. This study also underscores the subtle yet significant influence of solvent interactions on the reaction pathways of CO_2_ reduction by iron porphyrin complexes, highlighting the need for caution when extrapolating mechanisms from one solvent system to another.

## Author Contributions

**Andrew Howe**: investigation, writing – original draft, writing – review and editing, formal analysis. **Aude Salamé**: formal analysis, writing – review and editing, investigation. **Ulysse Garnier**: investigation, writing – review and editing, formal analysis. **Marc Robert**: conceptualization, formal analysis, funding acquisition, writing – review and editing, supervision. **Elodie Anxolabéhère‐Mallart**: conceptualization, formal analysis, funding acquisition, writing – review and editing, supervision. **Mun Hon Cheah**: conceptualization, funding acquisition, writing – original draft, writing – review and editing, formal analysis, supervision, data curation.

## Conflicts of Interest

The authors declare no conflicts of interest.

## Supporting information




**Supporting File**: The authors have cited additional references within the Supporting Information [13].

## Data Availability

The data that support the findings of this study are available from the corresponding author upon reasonable request.
