# Supplementary material for: CO2‐to‐CO Electrochemical Conversion With an Fe(I) Porphyrin Complex in Water
Source: Angew Chem Int Ed Engl. 2026 Aug 5;65(40):e7585384. doi: 10.1002/anie.7585384 (PMC13618285; doi:10.1002/anie.7585384)
Supplement: Supplementary file 1 [file ANIE-65-e7585384-s001.pdf]

CO_2-to-CO electrochemical conversion with an Fe(I) porphyrin complex in water

Andrew Howe[a], Aude Salamé[b,c], Ulysse Garnier[c], Marc Robert[c,d*], Elodie Anxolabéhère-Mallart[c,*], Mun Hon Cheah[a,*]

[a]Molecular Biomimetics, Department of Chemistry-Ångström, Uppsala University, 75120 Uppsala, Sweden

[b]Leiden Institute of Chemistry, Leiden University, P.O. Box 9502, 2300, RA, Leiden, the Netherlands

[c]Sorbonne Université, CNRS, Institut Parisien de Chimie Moléculaire (IPCM), F-75005 Paris, France

[d]Institut Universitaire de France (IUF), F-75005 Paris, France

Table of content

Experimental section. pH measurement of buffers, spectroelectrochemical measurements, electrochemical measurements and Gas Chromatography

Scheme S1. Structure of [(TPPS)Fe^{III}Cl].

Figure S1. UV-vis SEC data of 2 mM solution of [(pTMA)Fe^{III}Cl]Cl$_4$ dissolved in 0.2 M potassium phosphate buffer, 0.2 M KCl. 3 bar 100% CO_2.

Figure S2. Comparison of peak current of reduction wave at -0.90 V vs. Ag/AgCl under Ar and CO_2 atmosphere.

Figure S3. Comparison of IR spectra of [(pTMA)(Cl)Fe^{II}-CO]$^{3+}$ and [(pTMA)Fe^{II}-CO]$^{4+}$ that is formed in presence and absence of 0.2 M KCl

Supplementary Figure 4. Electrochemical data during bulk electrolysis of 2 mM solution of [(pTMA)Fe^{III}Cl] dissolved in 0.2 M potassium phosphate buffer, 0.2 M KCl.

Supplementary Figure 5. IR SEC data of 2mM of [(pTMA)Fe^{III}Cl]Cl$_4$ dissolved in 0.2 M potassium phosphate buffer under 3 bar 100% CO_2 (pH = 7) showing extended wavenumber range.

Supplementary Figure 6. UV-vis SEC data of 2 mM solution of [(TPPS)Fe^{III}Cl] dissolved in 0.2 M potassium phosphate buffer, 0.2 M KCl. 3 bar 100% Ar.

Supplementary Figure 7. UV-vis SEC data of 2 mM solution of [(TPPS)Fe^{III}Cl] dissolved in 0.2 M potassium phosphate buffer, 0.2 M KCl. 3 bar 100% CO_2.

Supplementary Figure 8. UV-vis SEC data of 2 mM solution of [(TPP)Fe^{III}Cl] dissolved in 0.2 M tetrabutylammonium hexafluorophosphate, 0.2 M H_2O, under 3 bar Ar and 3 bar CO_2.

Experimental section.

Chemicals. All chemicals used for aqueous buffer (Potassium dihydrogen phosphate, Potassium hydrogen phosphate, sodium bicarbonate) and electrolyte preparations (potassium chloride) were of highest commercially available grade from VWR, Merck or Sigma Aldrich. N,N-dimethylformamide (DMF) was purchased from Acros Organics (≥99.8%, Extra Dry, AcroSeal, over molecular sieves). Tetrabutylammonium hexafluorophosphate ($TBAPF_6$, ≥99%) was purchased from Sigma Aldrich.

Synthesis of $[(pTMA)Fe^{III}Cl]Cl_4$. $[(pTMA)Fe^{III}Cl]Cl_4$ was prepared according to the previously reported procedure.[1] The solid was characterized by cyclic voltammetry and UV-Visible spectroscopy, confirming the formation of pure $[(pTMA)Fe^{III}\text{-}Cl]$ species with four Cl^- counter-anions balancing the positive charges of the TMA groups.

pH measurement of buffer. The potassium phosphate buffer (0.2 M) containing KCl (0.2 M) and sodium bicarbonate buffer (0.2 M) containing KCl (0.2 M) were prepared, adjusted to pH 7.0 and stored in sealed containers before experiments. For SEC experiments conducted under 3 bar CO_2, the pH values of both buffers were estimated by bubbling CO_2 gas into a sample of prepared buffer electrolyte solution for approximately 30 mins and remeasure the pH value immediately after; the measured pH values are pH ≈ 6.

Spectroelectrochemical measurements. Scanning spectroelectrochemistry with UV-vis and FTIR spectroscopy were performed using a custom SEC setup based on reflection design that was describe in a previous publication.[1] The working electrode was a 3-mm-diameter glassy carbon disk. The counter-electrode was a graphite rod. The reference electrode was a homemade Ag/AgCl reference electrode (AgCl coated Ag wire in 3 M KCl solution in jacketed compartment). The working electrode is polished with aluminum oxide coated abrasive discs, starting from 9 µm to 0.3 µm, (FiberMet, Buehler) that is supported on a flat glass pane to obtain a flat and reflective surface to reflect IR or UV-vis light. A Biologic SP300 potentiostat was interfaced to either a Bruker 70v FTIR spectrometer or Avantes ULS2048L UV-vis spectrometer equipped with a Hamamatsu Photonics Xenon flash lamp (L9455-11). During scanning SEC experiments, a spectrum is collected at each potential step of a staircase CV scan with step size of 20 mV. The average scan rate is reported as 20 mV divided by time at each potential step for spectrum collection. Data analysis of SEC experiments were performed using Igor Pro version 9.0 from Wavemetrics.

Electrochemical measurements. Cyclic voltammograms (SI Figure 4) were recorded in a three-electrode cell. The working electrode was a 3-mm-diameter glassy carbon disk (0.071 cm^2) carefully polished with 1-µm and 0.25-µm diamond pastes for 1 minute each, then thoroughly sonicated in water for 2 minutes before use. The counterelectrode was a platinum grid separated from the cathodic compartment by a salt bridge (purchased from OrigaLys) and the reference electrode was an aqueous SCE electrode (KCl saturated, purchased from OrigaLys) also contained in a salt bridge. The controlled potential electrolysis was recorded using the same cell, reference electrode and counter electrode. The working electrode was a glassy carbon plate (2.96 cm^2) carefully polished with 1-µm and 0.25-µm diamond pastes for 1 minute each, then thoroughly sonicated in water for 2 minutes before use. These experiments were carried out either under saturated argon (Air Liquide, Argon Alphagaz 1 – Impurities : O_2 ≤ 2 ppm; CnHm ≤ 0.5 ppm; H_2O ≤ 3 ppm; CO_2 and CO ≤ 1 ppm) or carbon dioxide (Air Liquide, Carbon dioxide Alphagaz 1 – Impurities : O_2 ≤ 30 ppm; NO + NO_2 ≤ 2 ppm; H_2O ≤ 50 ppm; CO ≤ 5 ppm) atmosphere at room temperature after bubbling the gas for 30 minutes. Ohmic drop was compensated up to 80% of its value through the positive feedback compensation method implemented in the instrument. The instrument was a Metrohm Autolab PGSTAT302N potentiostat interfaced with Nova software.

Gas chromatography (GC). Gas chromatography analysis of gaseous samples were performed with a Agilent Technologies 7820A GC equipped with a thermal conductivity detector. CO was quantitatively detected using a CP-CarboPlot P7 capillary column (27.46 m

in length and 25 µm internal diameter). Temperature was held at 90 °C for the inlet, at 150 °C for the detector and 35 °C for the oven. The carrier gas was argon flowing at 8.0 mL.min^{-1} at constant pressure of 0.4 bar. Injection was performed via a 250 µL gas-tight (Hamilton) syringe. Conditions allowed detection of O_2, N_2, CO, and CO_2. Calibration curves of these gases were determined separately by injecting known quantities of pure gas.

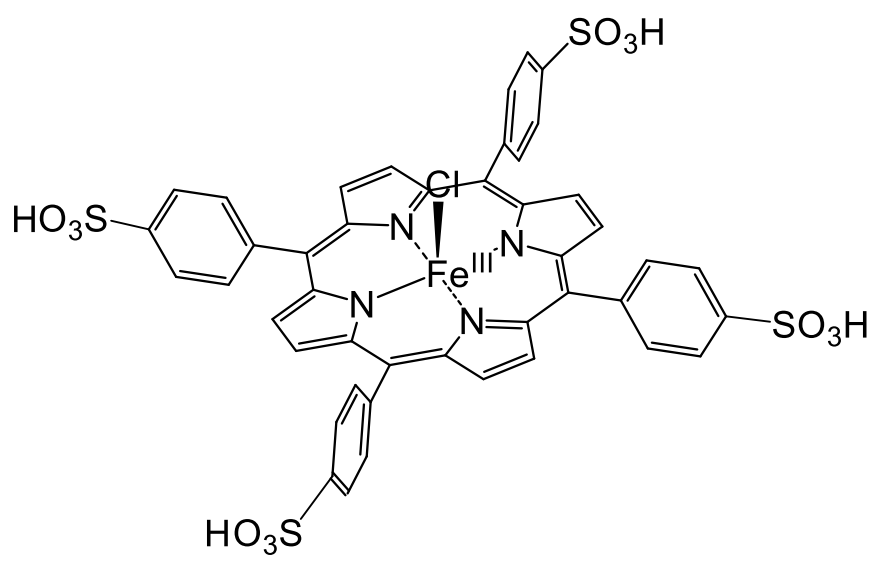

Supplemenrary Scheme 1. Structure of [(TPPS)Fe^{III}Cl].

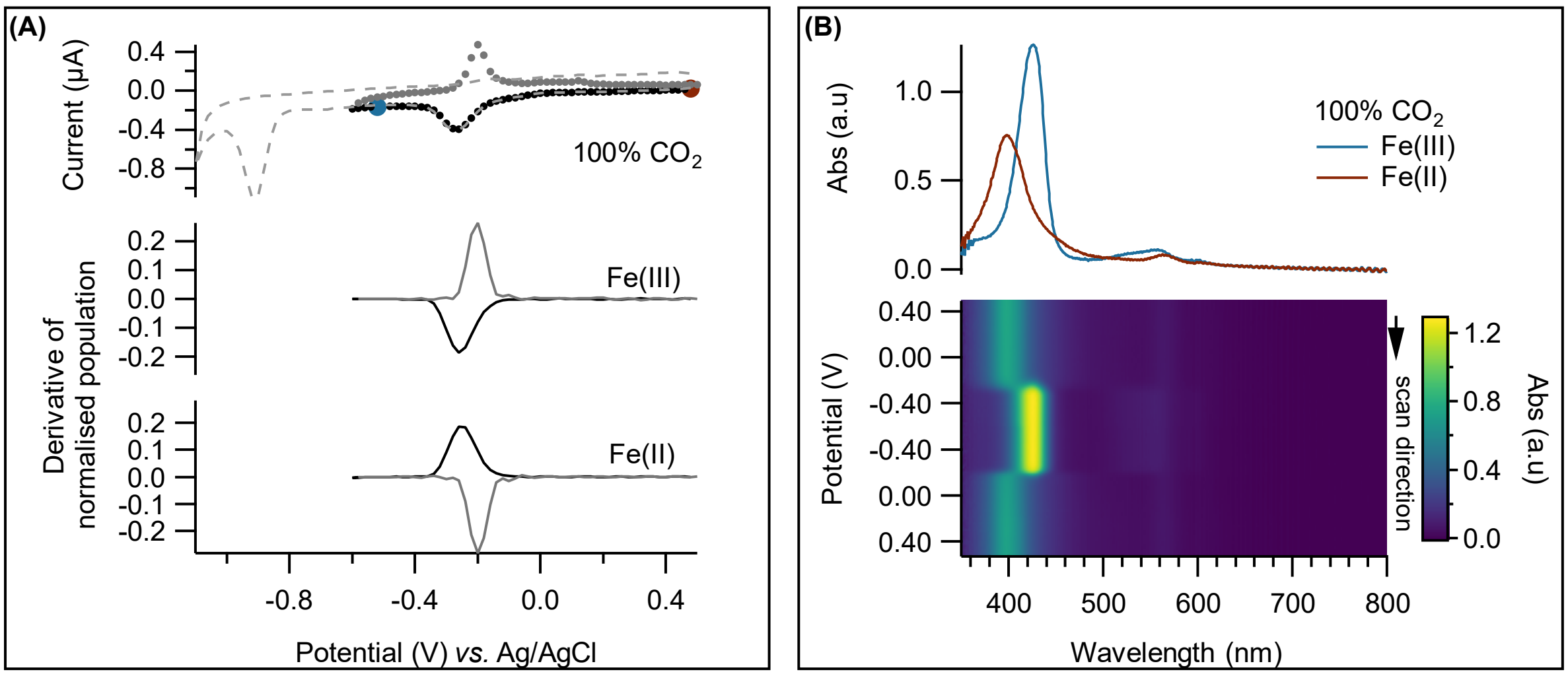

Figure S1. UV-vis SEC data of 2 mM solution of $[(pTMA)Fe^{III}Cl]Cl_4$ dissolved in 0.2 M potassium phosphate buffer, 0.2 M KCl. 3 bar 100% CO_2. The potential step is 20 mV and the average scan rate is 1 mV/s. Top panel of (A) shows current-voltage response during UV-vis SEC, cathodic scan direction in black and anodic scan direction in grey. Bottom panel of (A) shows the derivative of normalized population of [(pTMA)Fe] species, derived from deconvolution of UV-vis spectra. Top panel of (B) shows representative spectra recorded during UV-vis SEC where the applied potential is colour coded based on the matching colour of the large circular symbol in the corresponding panel (A). Bottom panels of (B) shows 2D-image plot of UV-vis spectra as function of applied potential.

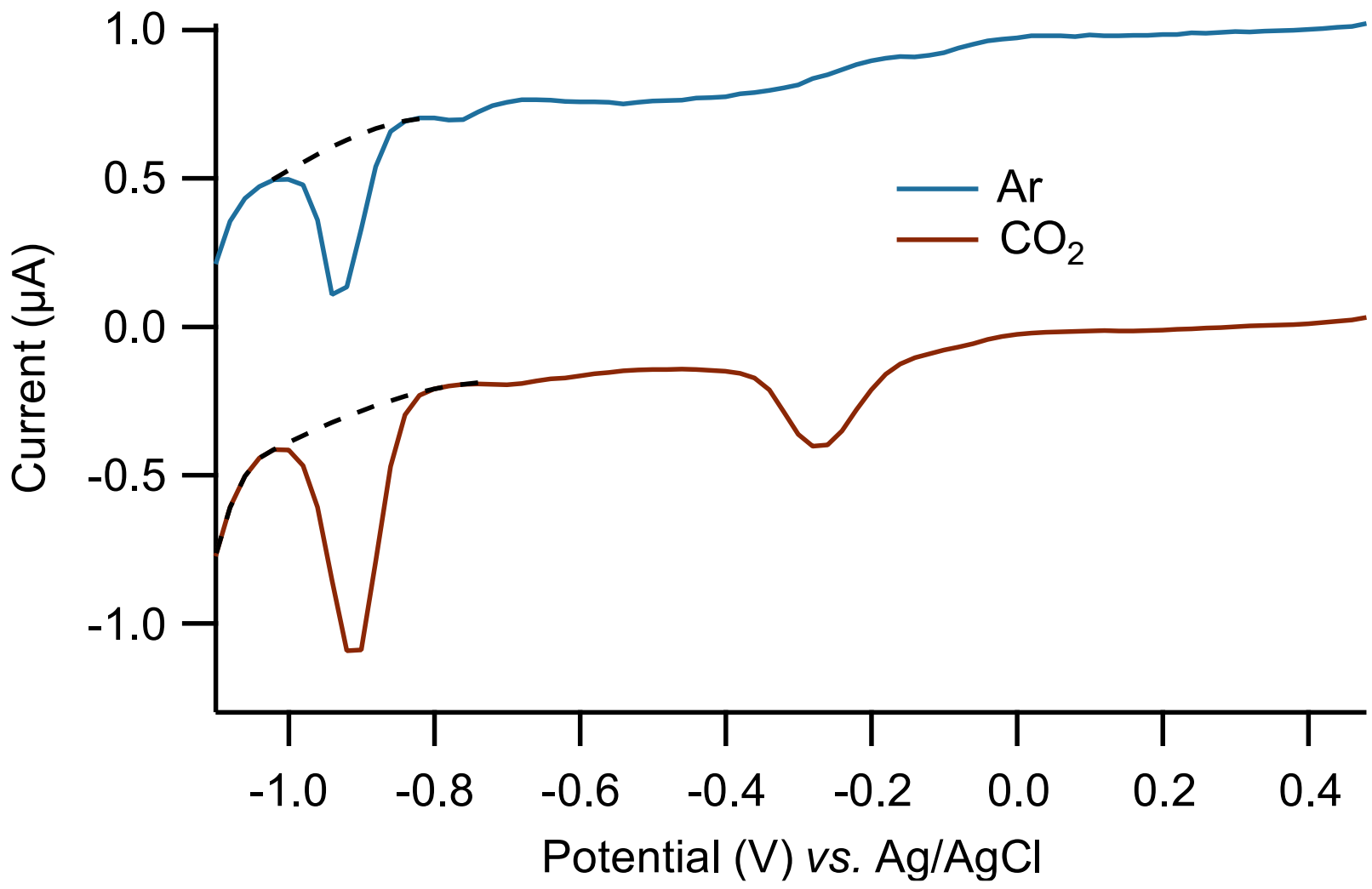

	Ar	CO_2
Film thickness	62.7 µm	57.3 µm
Integrated peak current (dimensionless)	0.0018	0.0033
Ratio CO_2/Ar integrated peak current (normalized film thickness)	(62.7/57.3)*(0.0033/0.018) = 2.01	

Figure S2. Comparison of peak current of reduction wave at -0.90 V vs. Ag/AgCl under Ar and CO_2 atmosphere. The film thickness is estimated based on analysis of interference fringes that is observable in the 650 nm to 700 nm region of the UV-vis spectra using the equation:

$$d = \frac{\Delta m}{2\sqrt{n^2 - \sin^2\theta}} \times \frac{1}{\frac{1}{\lambda_2} - \frac{1}{\lambda_1}}$$

d = film thickness
n = refractive index of water = 1.33
Θ = angle of incidence = 70.8°
Δm = number of peaks in wavelength range
λ = high and low peak wavelength

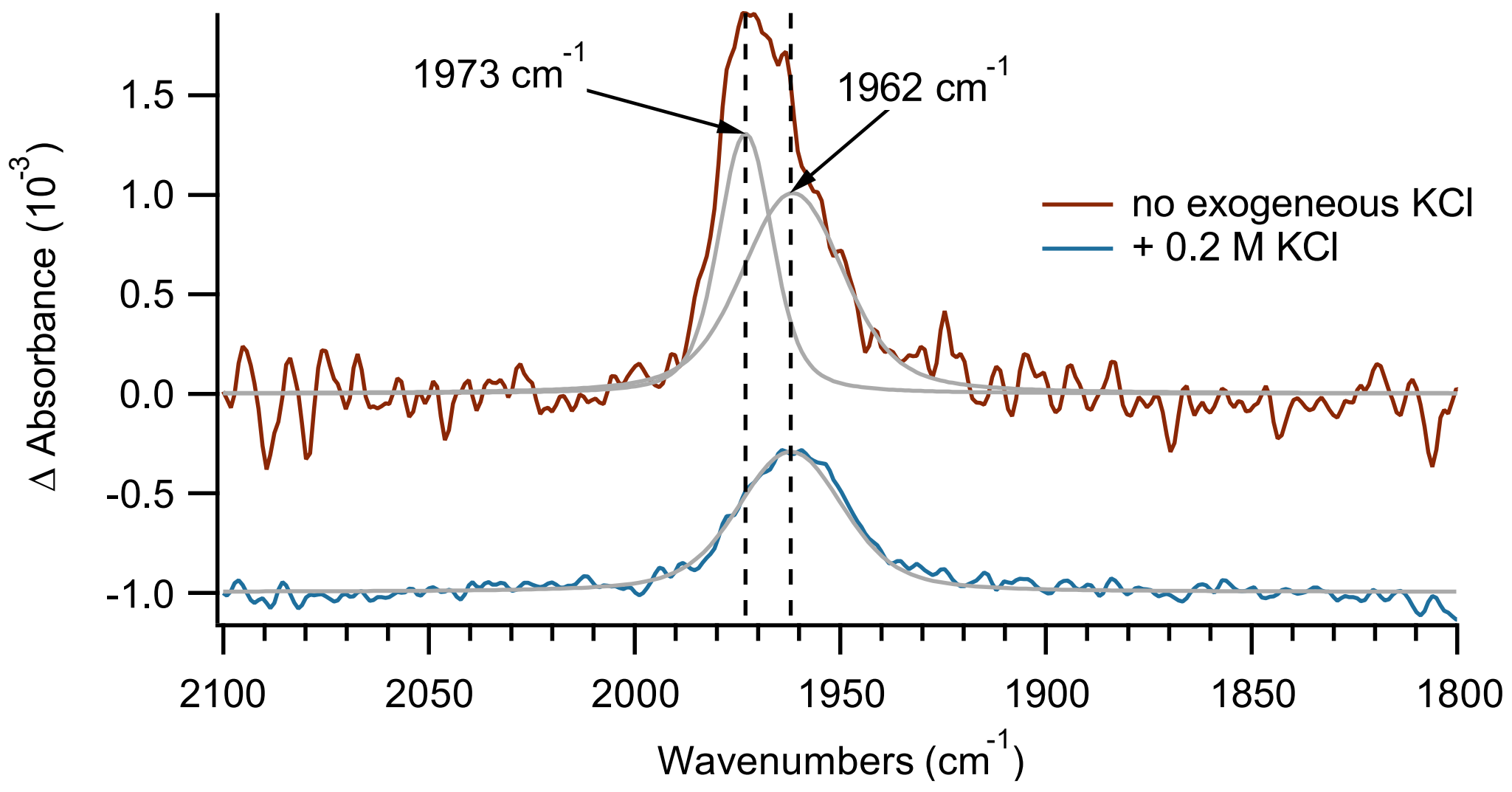

Figure S3. Comparison of IR spectra of $[(pTMA)(Cl)Fe^{II}\text{-}CO]^{3+}$ and $[(pTMA)Fe^{II}\text{-}CO]^{4+}$ that is formed in presence and absence of 0.2 M KCl. In absence of exogeneous KCl, the observed ν(CO) band is a symmetrical and can be fitted with two bands at 1973 cm^{-1} and 1962 cm^{-1} (grey lines). In presence of 0.2 KCl, the observed ν(CO) is symmetrical and can be satisfactorily fitted with a single band at 1962 cm^{-1}. The band at 1962 cm^{-1} is assigned to $[(pTMA)(Cl)Fe^{II}\text{-}CO]^{3+}$, while the band at 1973 cm^{-1} is assigned to $[(pTMA)Fe^{II}\text{-}CO]^{4+}$. Under conditions where no exogeneous KCl is added, a fraction of $[(pTMA)(Cl)Fe^{II}\text{-}CO]^{3+}$ exists because the parent complex $[(pTMA)(Cl)Fe^{III}]Cl_4$ added to solution contains 5 equivalents of Cl^-.

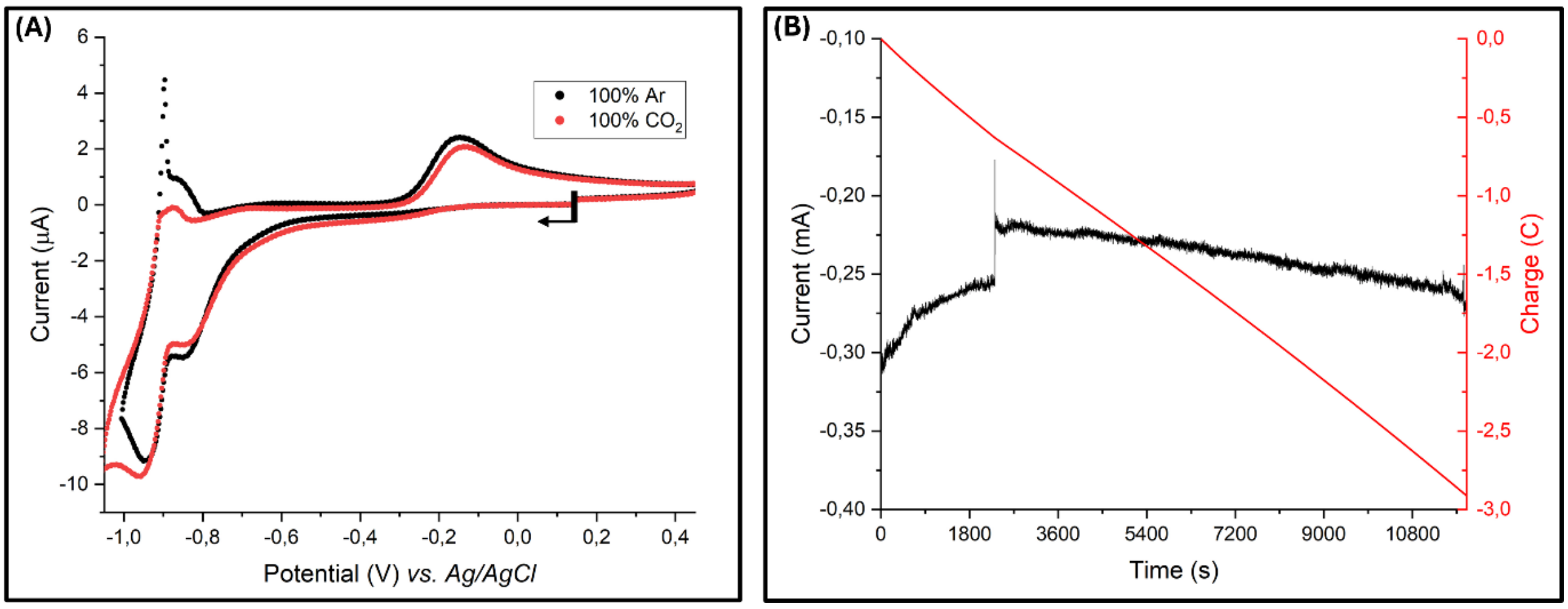

Figure S4. Electrochemical data of 2 mM solution of [(pTMA)Fe^{III}Cl] dissolved in 0.2 M potassium phosphate buffer, 0.2 M KCl. Panel (A) shows cyclic voltammograms under 1 bar 100% Ar atmosphere (Black points, pH 7.0) and 1 bar 100% CO_2 atmosphere (Red points, pH 6.4). (B) Control potential electrolysis performed under 1 bar 100% CO_2 atmosphere at -0.92 V vs. Ag/AgCl. (Black line) Current vs. time trace. (Red line) Charge vs. time trace. Following this experiment, no CO nor H_2 gas could be detected by GC.

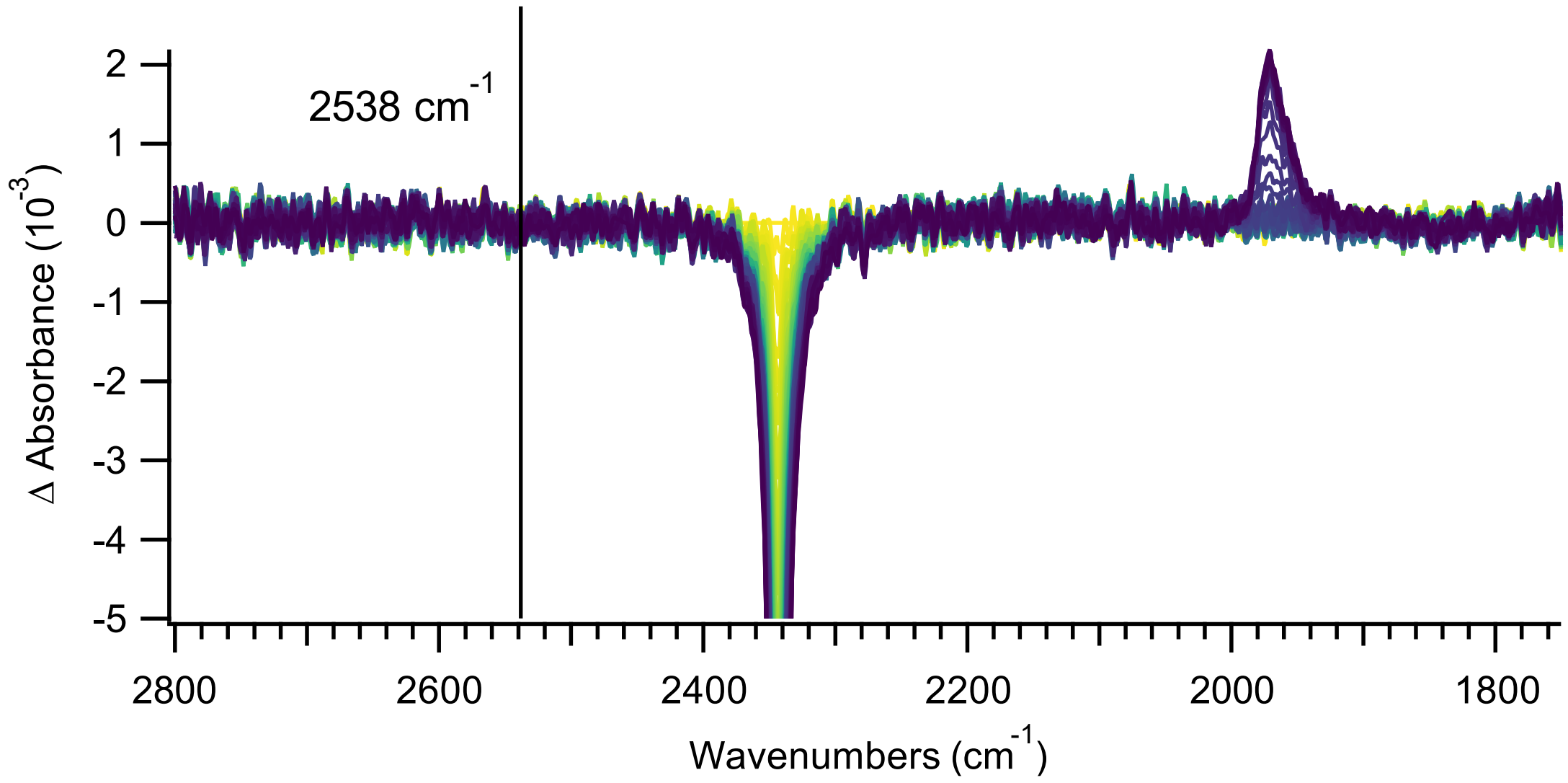

Figure S5. IR SEC data of 2mM of [(pTMA)Fe^{III}Cl]Cl_4 dissolved in 0.2 M potassium phosphate buffer under 3 bar 100% CO_2 (pH = 7). Applied potential from 0.19 V (light yellow) to -1.0 V (dark blue) vs. Ag/AgCl. Data reproduced from Figure 4 in main text, showing extended wavenumber range to examine if there is any spectral change near 2538 cm^{-1}.

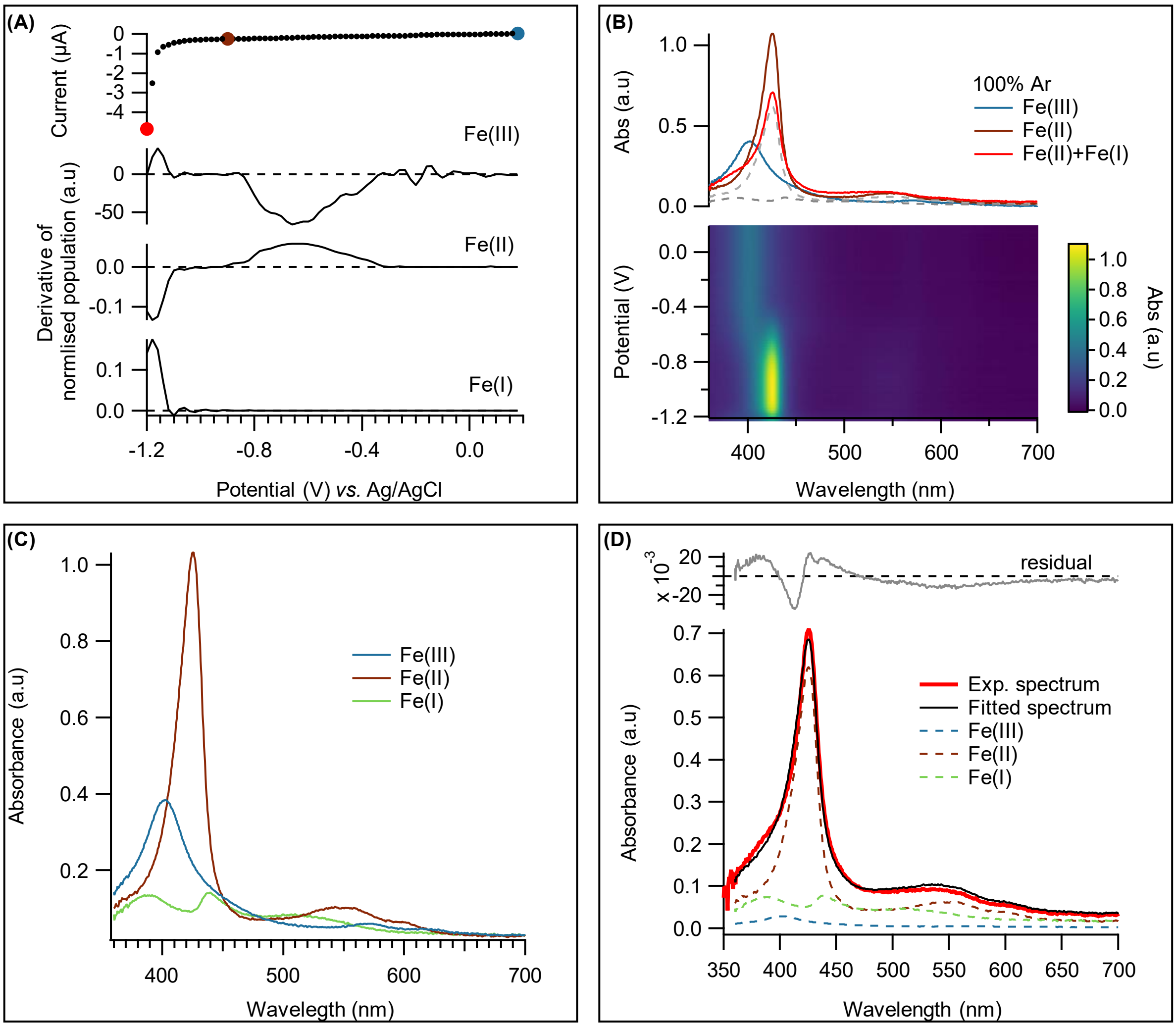

Figure S6. UV-vis SEC data of 2 mM solution of [(TPPS)Fe^{III}Cl] dissolved in 0.2 M potassium phosphate buffer, 0.2 M KCl. 3 bar 100% Ar. The potential step is 20 mV and the average scan rate is 1 mV/s. Top panel of (A) shows current-voltage response during UV-vis SEC, cathodic scan only. Bottom panel of (A) shows the derivative of normalized population of [(TPPS)Fe] species, derived from deconvolution of UV-vis spectra. Top panel of (B) shows representative spectra recorded during UV-vis SEC where the applied potential is colour coded based on the matching colour of the large circular symbol in the corresponding panel (A). Bottom panels of (B) shows 2D-image plot of UV-vis spectra as function of applied potential. Panel (C) shows representative spectra associated with Fe(III), Fe(II) and Fe(I) oxidation states of [(TPPS)Fe] species. Panel (D) shows deconvolution of spectrum recorded at -1.2 V, indicating a mixture of Fe(III), Fe(II) and Fe(I) present.

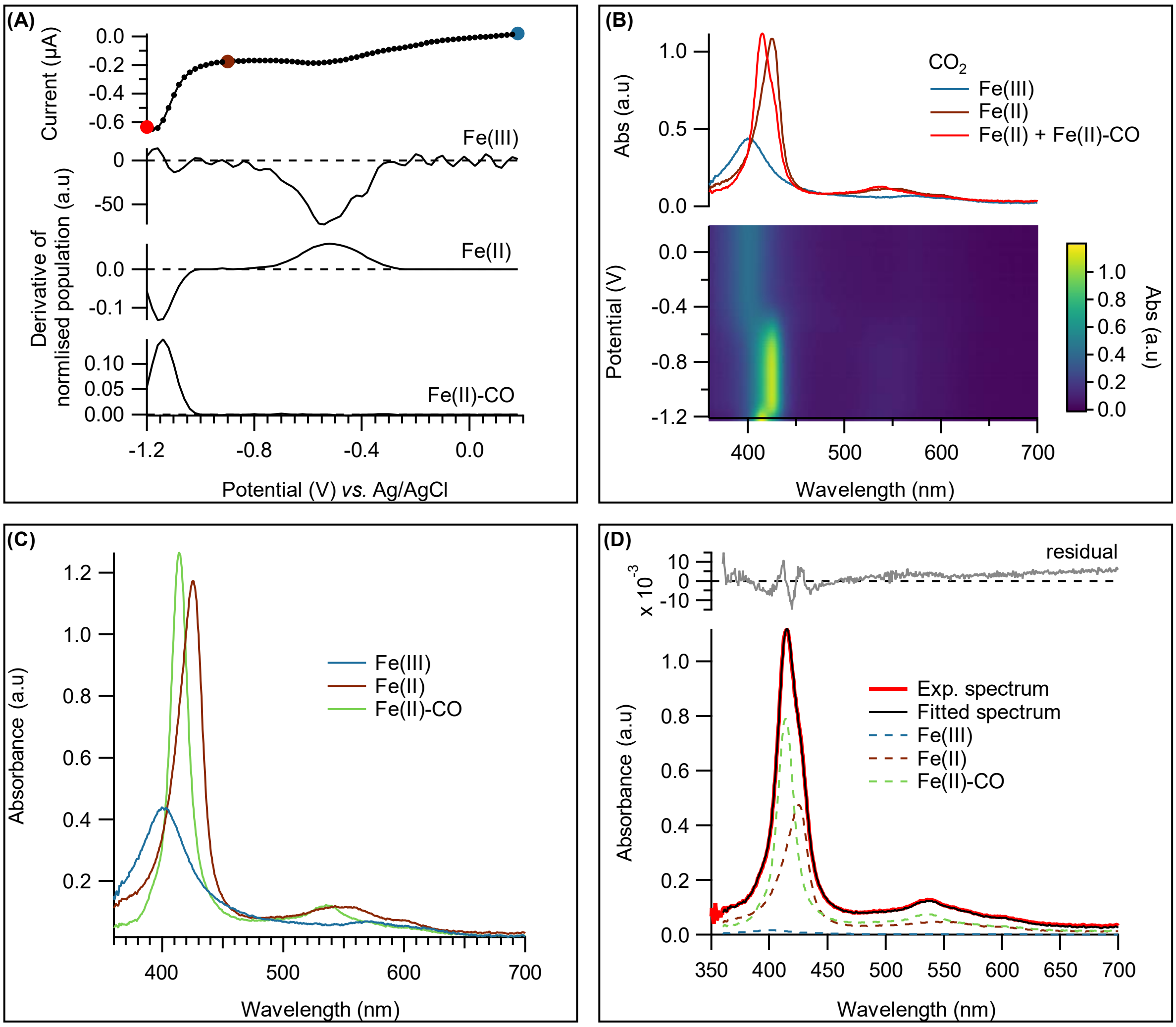

Figure S7. UV-vis SEC data of 2 mM solution of [(TPPS)Fe^{III}Cl] dissolved in 0.2 M potassium phosphate buffer, 0.2 M KCl. 3 bar 100% CO_2. The potential step is 20 mV and the average scan rate is 1 mV/s. Top panel of (A) shows current-voltage response during UV-vis SEC, cathodic scan only. Bottom panel of (A) shows the derivative of normalized population of [(TPPS)Fe] species, derived from deconvolution of UV-vis spectra. Top panel of (B) shows representative spectra recorded during UV-vis SEC where the applied potential is colour coded based on the matching colour of the large circular symbol in the corresponding panel (A). Bottom panels of (B) shows 2D-image plot of UV-vis spectra as function of applied potential. Panel (C) shows representative spectra associated with Fe(III), Fe(II) and Fe(II)-CO states of [(TPPS)Fe] species. Panel (D) shows deconvolution of spectrum recorded at -1.2 V, indicating a mixture of Fe(II) and Fe(II)-CO present.

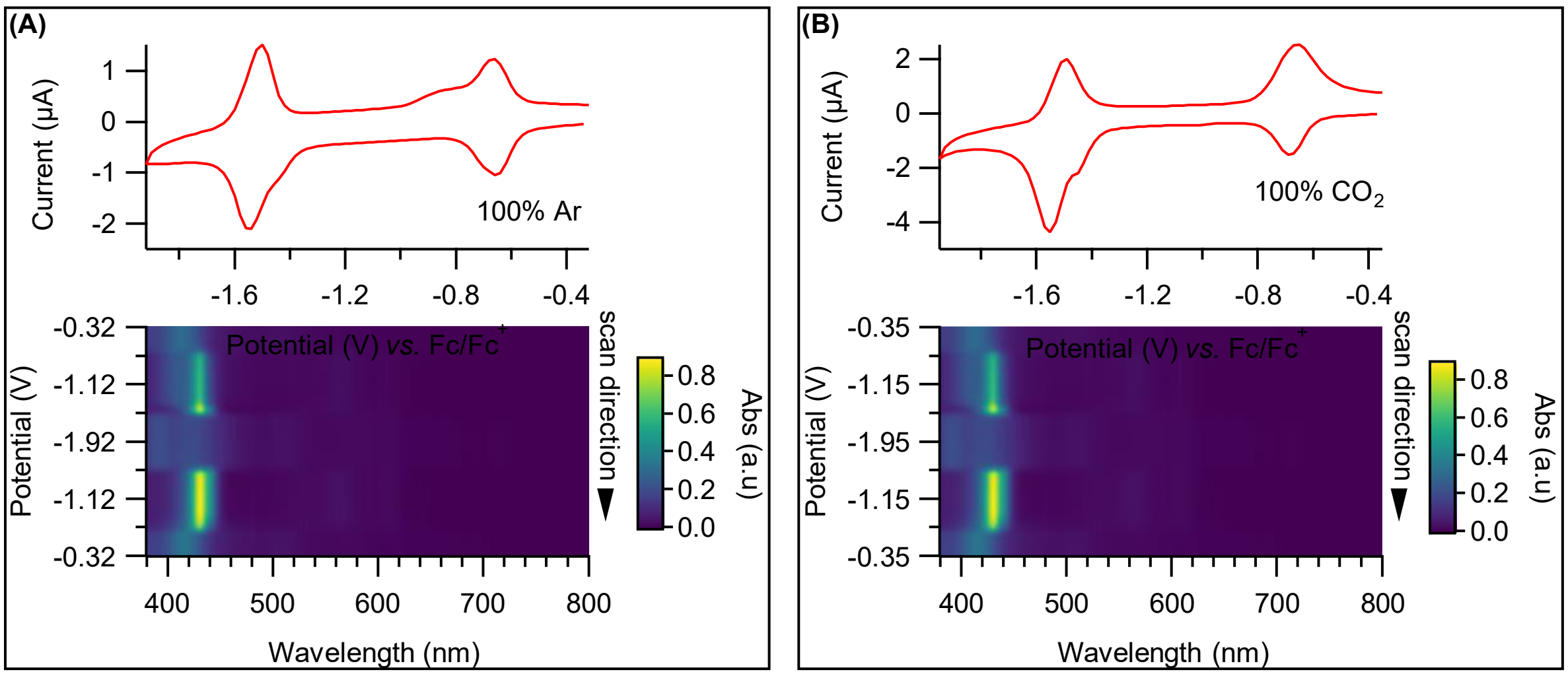

Figure S8. UV-vis SEC data of 2 mM solution of [(TPP)Fe^{III}Cl] dissolved in 0.2 M tetrabutylammonium hexafluorophosphate, 0.2 M H_2O. The potential step is 20 mV and the average scan rate is 10 mV/s. Top panel shows current-voltage response during UV-vis SEC, bottom panel shows 2D-image plot of UV-vis spectra as function of applied potential. (A) Experiment under 3 bar 100% Ar atmosphere. (B) Experiment under 3 bar 100% CO_2 atmosphere.

References

(1) Salamé, A.; Hon Cheah, M.; Bonin, J.; Robert, M.; Anxolabéhère-Mallart, E. Operando Spectroelectrochemistry Unravels the Mechanism of CO2 Electrocatalytic Reduction by an Fe Porphyrin. *Angewandte Chemie International Edition* **2024**, *63* (51), e202412417. https://doi.org/10.1002/anie.202412417.
